# Structure and Stability of Partial Dislocation Complexes in 3C-SiC by Molecular Dynamics Simulations

**DOI:** 10.3390/ma12183027

**Published:** 2019-09-18

**Authors:** Andrey Sarikov, Anna Marzegalli, Luca Barbisan, Francesco Montalenti, Leo Miglio

**Affiliations:** 1 Dipartimento di Scienza dei Materiali, Università degli Studi di Milano-Bicocca, via R. Cozzi 55, 20125 Milano, Italy; anna.marzegalli@unimib.it (A.M.); l.barbisan@campus.unimib.it (L.B.); 2 V. Lashkarev Institute of Semiconductor Physics, National Academy of Sciences of Ukraine, 45 Nauki avenue, 03028 Kiev, Ukraine; 3 L-NESS and Dipartimento di Fisica, Politecnico di Milano, via Anzani 42, 22100 Como, Italy; 4 L-NESS and Dipartimento di Scienza dei Materiali, Università degli Studi di Milano-Bicocca, via R. Cozzi 55, 20125 Milano, Italy; francesco.montalenti@unimib.it (F.M.); leo.miglio@unimib.it (L.M.)

**Keywords:** 3C-SiC films, extended defects, partial dislocations, dislocation complexes, molecular dynamics simulations

## Abstract

In this work, the structure and stability of partial dislocation (PD) complexes terminating double and triple stacking faults in 3C-SiC are studied by molecular dynamics simulations. The stability of PD complexes is demonstrated to depend primarily on the mutual orientations of the Burgers vectors of constituent partial dislocations. The existence of stable complexes consisting of two and three partial dislocations is established. In particular, two types of stable double (or extrinsic) dislocation complexes are revealed formed by two 30° partial dislocations with different orientations of Burgers vectors, or 30° and 90° partial dislocations. Stable triple PD complexes consist of two 30° partial dislocations with different orientations of their Burgers vectors and one 90° partial dislocation, and have a total Burgers vector that is equal to zero. Results of the simulations agree with experimental observations of the stable PD complexes forming incoherent boundaries of twin regions and polytype inclusions in 3C-SiC films.

## 1. Introduction

Properties of cubic silicon carbide (3C-SiC) that combine a large band gap value as compared to that of Si (2.35 vs. 1.1 eV) [1], high bulk and channel electron mobility (up to 1000 and 250 cm^2^/Vs, respectively) [2,3], and low trap density at the 3C-SiC/SiO_2_ interfaces of the metal-oxide-semiconductor structures [4] make it one of the most suitable candidates for power MOSFET devices in the range of breakdown voltages below 800 V for hybrid electric vehicles, air conditioning, and LED lighting system applications [5,6]. The absence of reliable growth technology for 3C-SiC bulk crystals forces 3C-SiC films to be grown heteroepitaxially on Si substrates, which raises quality issues, mainly originating from the lattice mismatch between the dissimilar substrate and film materials (about 20%), as well as the difference in their thermal expansion coefficients [7]. Various kinds of extended defects, such as microtwins, antiphase boundaries, and stacking faults (SFs), develop at the Si/3C-SiC interface, induced by stress release processes, and propagate into the 3C-SiC films during growth. Concentrations of these defects decrease with the increase of the film thickness by a self-annihilation mechanism; some of them (e.g., microtwins) may even completely disappear in very thick 3C-SiC layers [6,8]. On the other hand, additional generation of stacking faults takes place during 3C-SiC film growth due to local strain fields and faults in the atomic arrangement, turning the saturation of the bulk density of this type of extended defects to the value of about 10^4^ cm^−1^ [6].

Stacking faults are the most frequent extended defects in 3C-SiC, which have high electrical activity [9,10]. In addition to single stacking faults, double and triple SFs are quite abundant, resulting in the inclusions of hexagonal 4H- and 6H-SiC polytypes with band characteristics different from those of 3C-SiC. Stacking faults are inseparable from the 30° and 90° Shockley partial dislocations, which decorate them at the border with bulk crystal. In general, partial dislocations should be given particular attention as they are important components of the microstructure of not only bulk semiconductors, like the 3C-SiC considered in this work, but also of advanced material structures, such as 2d materials, crucially influencing their conductivity and mechanical characteristics [11,12].

In 3C-SiC, multiple stacking faults can be terminated by dislocation complexes formed by interaction of individual partial dislocations. Dislocation complexes consisting of two and three partial dislocations have been observed experimentally at multiple stacking fault boundaries by high-resolution transmission electron microscopy (HRTEM) investigations [13,14,15,16]. They may have a recombination activity induced by deep levels in the band gap of 3C-SiC due to the rearrangement of atoms as compared to the perfect crystal lattice and the formation of dangling bonds in the complex cores. Understanding of the stability and evolution dynamics of SFs and terminating them partial dislocation complexes is a prerequisite for the elaboration of the technology of their elimination and improvement of the electrical characteristics of 3C-SiC layers.

In our previous publication [17], we demonstrated that molecular dynamics (MD) simulations with analytical bond order (ABOP) [18] and Vashishta [19] potential applied synergetically are an efficient tool to model the behavior of extended defects, such as stacking faults and dislocations, in cubic silicon carbide. The Vashishta potential is preferred for the simulation of large-scale defect evolution, characterized in terms of the long-distance interaction of dislocations and stacking fault transformations. On the other hand, ABOP enables the more correct reproduction of atomic configurations of dislocation cores, which are predicted with a smaller accuracy with Vashishta potential due to the repulsion between the atoms of the same types imposed by its mathematical structure.

In this paper, we applied MD simulations with both analytical bond order and Vashishta potential to model the dynamics of the 30° and 90° Shockley partial dislocations and their complexes terminating double and triple stacking faults in 3C-SiC. The interaction between the partial dislocations as a function of the mutual orientations of their Burgers vectors, as well as the influence of this interaction on the stability of partial dislocation complexes, were studied. We demonstrated the existence of stable complexes formed by two and three partial dislocations. The atomic configurations of the stable partial dislocation complexes in 3C-SiC provided by MD simulations can be further used as an input for the ab initio study of their electric properties and thus for better prediction of the electric characteristics of 3C-SiC layers.

## 2. Methodology

The dynamics of the stability of partial dislocation complexes in 3C-SiC have been studied by molecular dynamics simulations using two empirical potentials that describe the interaction between the Si and C atoms. Namely, analytical bond order [18] and Vashishta potentials [19] have been applied as the most appropriate ones for the description of the behavior of extended defects in 3C-SiC material, in accordance with the results obtained in our previous publication [17]. As demonstrated in [17], MD simulations with both mentioned potentials predict qualitatively equal scenarios of extended defect evolution. At this, the Vashishta potential enables observation of the evolution of defects at much shorter time scales as compared to that obtained with ABOP, and is therefore used to greatly reduce the computation effort of simulations. In its turn, the ABOP provides a more correct description of the formation of local bonding and atomic configurations of dislocation core structures. It is used for verifying the predictions made by the Vashishta potential concerning the extended defect evolution and each time when the atomic structures of the dislocation cores are studied. With respect to this, performing MD simulations with both ABOP and Vashishta potential and comparing obtained results is a good strategy to study the dynamics of partial dislocations and dislocation complexes.

MD simulations have been performed using the Large-scale Atomic/Molecular Massively Parallel Simulator (LAMMPS) code [20] in the Nose-Hoover thermostat regime at the temperature of 1800 K. The value of the time step has been chosen equal to 0.3 fs based on the energy conservation of the simulation system in the course of preliminary simulation runs. To avoid the temperature shock effect on the 3C-SiC system, the temperature was increased from 300 to 1800 K during 1.5 ps of simulated time. Prior to the temperature increase, the system was allowed to relax using the LAMMPS energy minimization procedure with the conjugate gradient algorithm. MD simulations were carried out applying the periodic boundary conditions on all sides of the simulation cell bounded by the planes with orientations $\left[1\bar{1}\bar{2}\right]$, $\left[1\bar{1}1\right]$, and [110] in the directions of the axes *X*, *Y*, and *Z*, respectively. The cell contained 65,280 atoms and was sized as $30\times \frac{\sqrt{6}}{6}4a$ × $17\times \frac{\sqrt{3}}{3}6a$ × $2\times \frac{\sqrt{2}}{2}2a$, where *a* is the 3C-SiC lattice constant slightly dependent on the MD potential used. The dimensions of the simulation cell were chosen as the minimum ones the increase of which made already no effect on the behavior of the dislocations and dislocation complexes during probe MD simulations. Analysis of the defect structures in the 3C-SiC systems obtained at various time steps was performed using the Open Visualization Tool (OVITO) software (version 2.9.0) [21].

Partial dislocations were inserted into the $\left[1\bar{1}1\right]$ slip planes perpendicular to the *Y* axis, with the directions of the dislocation lines coinciding with the *Z* axis direction of the initial simulation cell. To form the dislocations, all the cell atoms were displaced by the vectors, with components calculated in the framework of the dislocation theory [22] as follows:

(1) $$u_{x}=\frac{b_{edge}}{2\pi }\left[\tan^{-1}\frac{y}{x}+\frac{xy}{2(1-\nu )(x^{2}+y^{2})}\right]$$

(2) $$u_{y}=-\frac{b_{edge}}{2\pi }\left[\frac{1-2\nu }{4(1-\nu )}\ln(x^{2}+y^{2})+\frac{x^{2}-y^{2}}{4(1-\nu )(x^{2}+y^{2})}\right]$$

(3) $$u_{z}=\frac{b_{screw}}{2\pi }\tan^{-1}\frac{y}{x}$$

where *b_edge_* and *b_screw_* are the edge and the screw components of the dislocation Burgers vector, respectively, ν = 0.25 is the 3C-SiC Poisson ratio [23], and *x*, *y*, and *z* are the atom coordinates, respectively.

**Figure 1:**
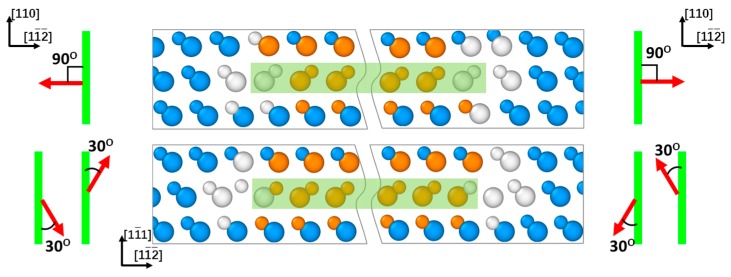
Atomic configurations of the dislocation dipoles consisting of Si- and C-terminated 90° (top panel) and C- and Si-terminated 30° (bottom panel) Shockley partial dislocations viewed in the direction [110]. Left and right panels show schematic representations of the dislocation Burgers vectors with respect to the dislocation lines viewed perpendicular to the $\left[1\bar{1}1\right]$ plane. Highlighted are the stacking faults formed between the partial dislocations in the dipoles. Vertical lines in the left and right panels correspond to the dislocation lines, and the arrows indicate possible directions of the dislocation Burgers vectors, respectively.

A dislocation dipole consisting of a pair of 30° or 90° PDs with opposite Burgers vectors separated by a stacking fault naturally formed between them was inserted into each selected $\left[1\bar{1}1\right]$ slip plane. The total Burgers vector of the partial dislocations in each dislocation dipole is therefore equal to zero. Possible configurations of the dislocation dipoles and corresponding dislocation Burgers vectors are shown in Figure 1. As can be seen from this figure, each dipole contained PDs terminated by Si and C atoms on the opposite sides of the stacking fault. At this, Si-terminated 90° and C-terminated 30° dislocations are located on the same stacking fault side (left one in Figure 1), while C-terminated 90° and Si-terminated 30° PDs are on the opposite SF side (right one in Figure 1), respectively. The dislocation dipoles were inserted into two or three consecutive $\left[1\bar{1}1\right]$ planes in the *Y* axis direction with all possible orders of constituting PDs to study the influence of the mutual orientations of the dislocation Burgers vectors on their interaction and stability of double or triple dislocation complexes.

## 3. Results

In this section, we present the results of the molecular dynamics simulations of the interaction of partial dislocations (PDs) terminating stacking faults, which for certain combinations of the Burgers vectors leads to the formation of stable double and triple PD complexes. It should be noted that all MD simulations were performed with both ABOP and Vashishta potential having resulted in equivalent dynamics of partial dislocations and dislocation complexes, the difference being only the smaller time scale with Vashishta potential as compared to that with ABOP. In addition, the results are presented here only for the Si-terminated 30° and C-terminated 90° PDs shown in the right panel of Figure 1 that bound the stacking faults located in the same $\left[1\bar{1}1\right]$ semi-planes with respect to the dislocation line. It should be noted, however, that the same qualitative results are obtained considering instead the interactions between the Si-terminated 90^O^ and C-terminated 30° partial dislocations.

### 3.1. Extrinsic Stacking Faults and Partial Dislocation Complexes

In the framework of the approach set forth above, we consider here the double dislocation complexes comprising two partial dislocations located in consecutive $\left[1\bar{1}1\right]$ planes, with any combination of the Burgers vectors of those presented in the right panel of Figure 1. Depending on the mutual orientations of the dislocation Burgers vectors, these complexes may be stable and form spontaneously as a result of PD interaction, or unstable as will be seen in more detail below.

**Figure 2:**
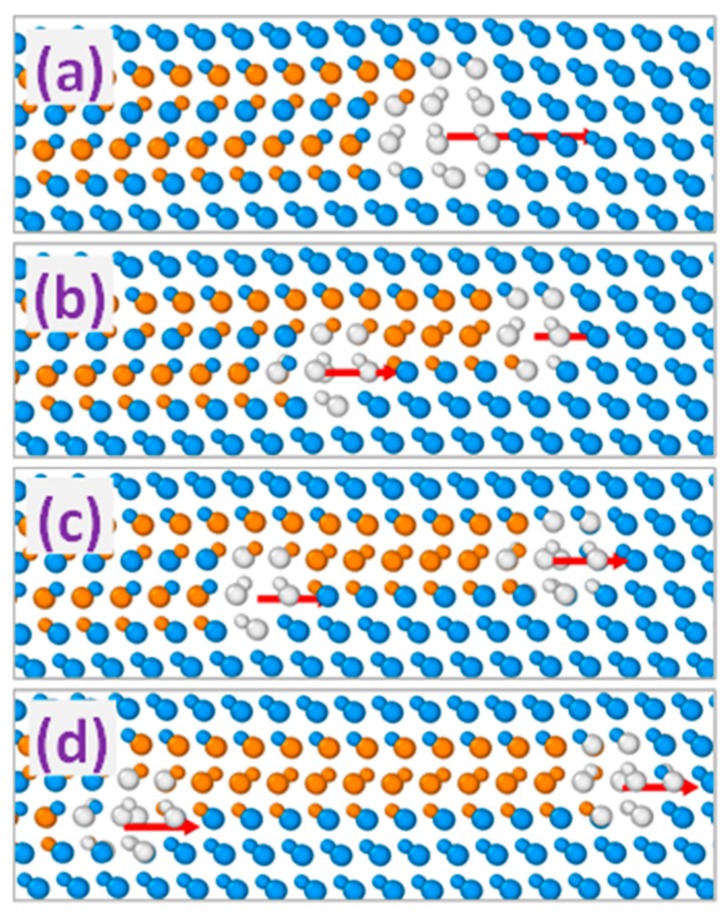
MD simulated evolution of the dislocation complex formed by a pair of 90° Shockley partial dislocations with equally oriented Burgers vectors. Simulated time: (**a**): 0, (**b**): 10, (**c**): 20, and (**d**): 50 ps, respectively. Arrows indicate the directions of the Burgers vectors of complex (**a**) and single dislocations (**b**–**d**).

Figure 2 shows MD simulation snapshots of the evolution of two 90° partial dislocations with equal Burgers vectors initially forming a double dislocation complex. The results presented in Figure 2b–d demonstrate that such a complex is unstable and dissociates into individual 90° dislocations that tend to separate from each other due to the repulsion between them. The analogous trend is observed for double dislocation complexes formed by 30° PDs with equal Burgers vectors, the difference being in slower separation kinetics due to the smaller motion velocity of 30° partials as compared to that of 90° ones [17].

**Figure 3:**
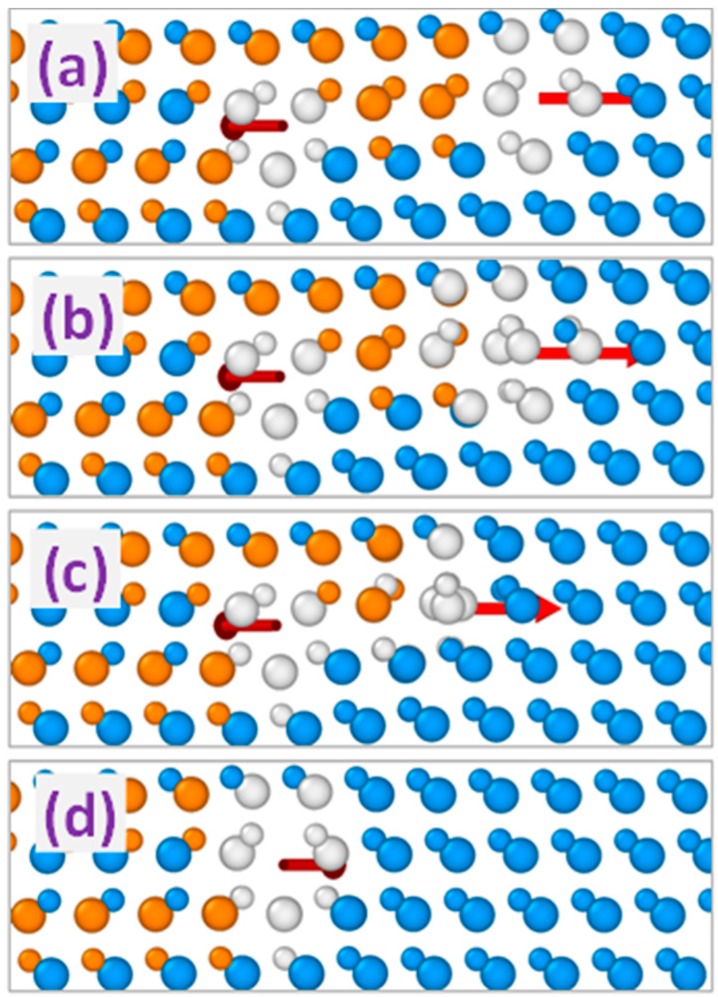
MD simulated evolution of the interaction of 30° and 90° partial dislocations forming the 30° extrinsic partial dislocation. Simulated time: (**a**): 0, (**b**): 20, (**c**): 50, and (**d**): 100 ps, respectively. Arrows indicate the directions of the Burgers vectors of the partial dislocations (**a**–**c**) and the dislocation complex (**d**).

**Figure 4:**
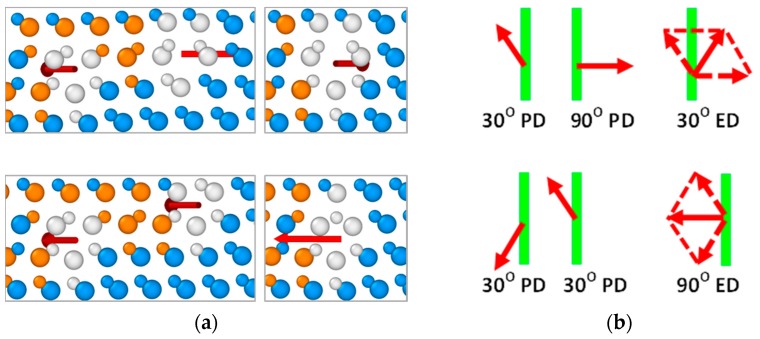
(**a**) MD simulation snapshots and (**b**) schematic top views of the formation of stable 30° (top images) and 90° (bottom images) extrinsic partial dislocations in 3C-SiC. Arrows indicate the directions of the dislocation and dislocation complexes Burgers vectors. Vertical lines in part (**b**) correspond to the dislocation lines.

Other possible variants of double dislocation configurations comprise interaction of 90° and 30° partial dislocations or two 30° partials with opposite directions of the screw components of their Burgers vectors (projections of the Burgers vectors on the dislocation lines). Unlike the previously considered repulsion between the PDs with equal Burgers vectors, the interaction within these dislocation pairs is attractive so that they form double dislocation complexes, as exemplified in Figure 3 for the 30° and 90° partial pair. The Burgers vector of the resulting double dislocation complex obtained as the sum of the Burgers vectors of composing partial dislocations corresponds for the latter case to that of the 30° partial as shown schematically in the top part of Figure 4. On the other hand, interacting 30° partials with opposite screw components of the Burgers vectors form the complex with the Burgers vector corresponding to that of the 90° partial dislocation (see the bottom part of Figure 4). Obtained PD complexes terminate double (or extrinsic) stacking faults so that they are referred to as 30° and 90° extrinsic partial dislocations, respectively. According to the MD simulations, both 30° and 90° extrinsic dislocations are stable in the sense that no change of their structures is observed after their formation as a result of simulated annealing at the temperature up to 1800 K for duration up to 2 ns. In addition to configuration stability, no change in the positions of extrinsic partial dislocations of both types has been observed as a result of MD simulations at the temperature and duration mentioned above.

### 3.2. Triple Stacking Faults and Partial Dislocation Complexes

**Figure 5:**
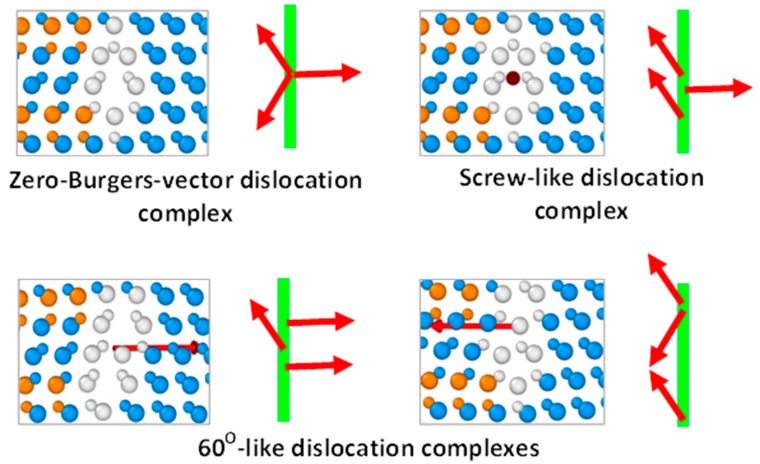
Examples of the atomic configurations of triple PD complexes in 3C-SiC viewed in the [110] direction together with schematic representations of dislocation Burgers vectors with respect to the dislocation lines viewed perpendicular to the $\left[1\bar{1}1\right]$ plane. Zero-Burgers-vector complex is stable, while other complexes experience dissociation into double PD complex and single partial dislocation during annealing. Arrows indicate the directions of the Burgers vectors of the complexes as well as complex composing partial dislocations. Vertical lines correspond to the dislocation lines.

Similar to the extrinsic partial dislocations, PD complexes consisting of three partial dislocations with any combination of the Burgers vectors presented in Figure 1 can be considered. However, we will not address dislocation complexes consisting of only PDs with equal Burgers vectors, in view of their easily predicted instability due to the repulsion between such dislocations (see previous section). Other possible combinations of the Burgers vectors of partial dislocations in the triple dislocation complexes, together with respective exemplary structures of these complexes before annealing viewed in the direction [110], are presented in Figure 5. As can be seen from this figure, depending on the dislocation set, triple PD complexes with zero total Burgers vectors, as well as with the Burgers vectors corresponding to the screw and 60° perfect dislocations, can be formed. At this, 60°-like dislocation complexes may be formed by two PD sets, namely 90°-30°-90° and 30°-30°-30° dislocations, the directions of the Burgers vectors of which are shown in the bottom part of Figure 5. It should also be mentioned that since the triple PD complexes with zero and screw-like Burgers vectors differ by only the direction of the screw component of one of the constituent 30° partial dislocations, their structures viewed along the dislocation lines are indistinguishable.

MD simulations of the dynamics of triple dislocation complexes have demonstrated the dependence of their stability on the combination of the Burgers vectors of individual partial dislocations composing them. In particular, only zero-Burgers-vector triple dislocation complexes were found stable. No change of the structures of these complexes was observed for simulated annealing temperatures up to 1800 K and simulated duration up to 2 ns for any stacking order of the composing partial dislocations. Moreover, MD simulations with the parameters mentioned above revealed the preservation of the positions of zero-Burgers-vector dislocation complexes, similar to the extrinsic partial dislocations considered in the previous section.

**Figure 6:**
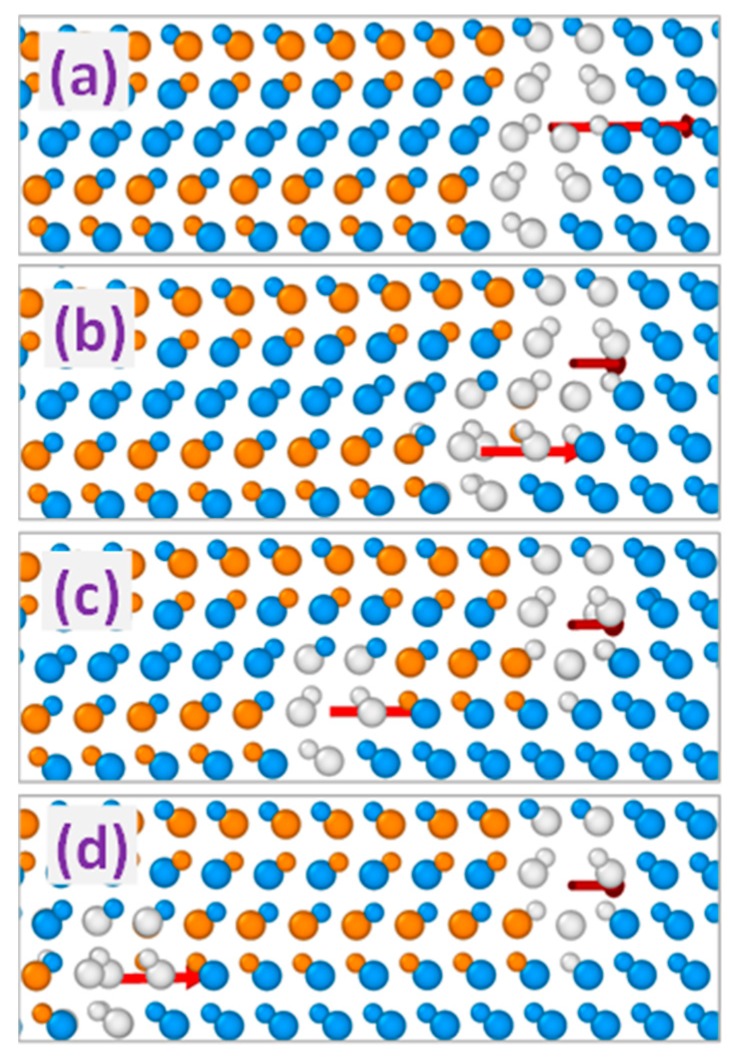
MD simulated evolution of the 60°-like triple dislocation complex formed by 90°-30°-90° partial dislocation sequence. Simulated time: (**a**): 0, (**b**): 30, (**c**): 60, and (**d**): 100 ps, respectively. Arrows indicate the directions of the Burgers vectors of the triple dislocation complex (**a**) as well as double dislocation complex and 90° partial dislocation (**b**–**d**).

On the opposite, all the triple PD complexes with non-zero total Burgers vectors were found to be unstable using MD simulations. The evolution of such complexes during annealing consists in the separation of a single partial dislocation leaving one of the stable 90° or 30° extrinsic partial dislocations considered in the previous section. In Figure 6, this process is exemplified for the 60°-like dislocation complex built by the 90°-30°-90° PD sequence: Dissociation produces stable 30° extrinsic dislocation and 90° partial that tends to move away. Dissociation kinetics of the non-zero-Burgers-vector triple dislocation complexes obtained during molecular dynamics simulation with both used potentials is dependent on the alignment of partial dislocations in the initial structure of the complex, indicating the activated character of this process similar to the dissociation of perfect 60° dislocation into the pair of 90° and 30° partials considered in our previous publication [17].

## 4. Discussion

Results of the experimental investigations evidence that concentrations of stacking faults remain at the level of about 10^4^ cm^–1^ even in very thick (several tens of microns) 3C-SiC films epitaxially grown on Si substrates and this value is extremely difficult to reduce [6]. SF generation during 3C-SiC growth takes place as a means to release local strain fields or due to the faults in the atomic arrangements, assisted by the small value of the SF formation energy [24]. In addition to single stacking faults, multiple SFs creating twin regions and SiC polytype inclusions are formed. Each stacking fault has an interface to the rest of the 3C-SiC crystal via 30° or 90° Shockley partial dislocations, which determine the SF evolution during technological processing of 3C-SiC films.

**Figure 7:**
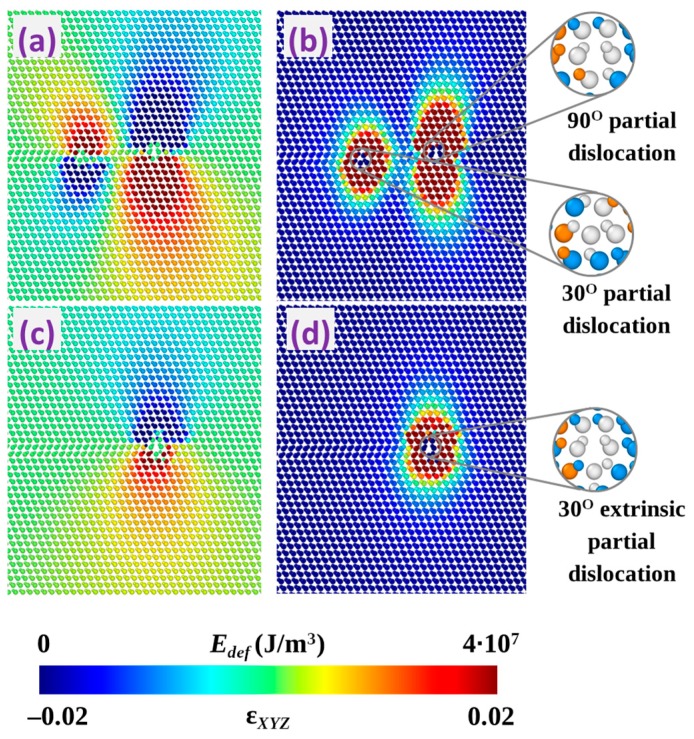
Distribution of volumetric strain (**a**,**c**) and elastic energy density (**b**,**d**) in 3C-SiC cell introduced by separated 30° and 90° partial dislocations located in consecutive $\left[1\bar{1}1\right]$ lattice planes (**a**,**b**) and 30° extrinsic partial dislocation formed as a result of their interaction (**c**,**d**).

Mobile individual partial dislocations terminating stacking faults interact by repulsing or attracting each other depending on the characteristics of the strain fields they produce. Figure 7 shows an example of the evolution of the deformation during formation of 30° extrinsic partial dislocation by attractive interaction of 90° and 30° partials. In this figure, the color map of the volumetric strain (ε*_xyz_*), i.e., the trace of strain tensor matrix, and the elastic energy density (*E_def_*) is presented. It is clearly seen that the values of ε*_xyz_* induced by individual partial dislocations have opposite signs in the considered case (see Figure 7a). The deformation noticeably decreases after formation of the double dislocation complex, as can be observed both in terms of energy and volumetric strain (see Figure 7c,d), such a decrease being just the driving force for the formation of the stable partial dislocation complexes obtained by MD simulations. We also verified that in the case of unstable complexes, such as the one formed by two identical 90° partial dislocations as illustrated in Figure 2, both the energy and the volumetric strain is reduced by the partial dissociation and the increase of the distance between them. Moreover, the highest stability is obtained for the zero-Burgers-vector triple dislocation complexes that introduce no strain in the 3C-SiC lattice apart from the local region of the complex core.

The interfaces of the regions composed of stacking faulted planes with respect to the surrounding crystal are stabilized by the formation of double (30° and 90° extrinsic partial dislocations) and triple (zero-Burgers-vector) dislocation complexes. At this, shifts of these interface boundaries also become suppressed. As mentioned above, we observed no motion of all the stable double and triple dislocation complexes in our MD simulations performing simulated annealing at temperatures up to 1800 K for a simulated duration of up to 2 ns. This indicates at least orders of magnitude smaller complex mobilities as compared to those of individual partial dislocations. As a consequence, twined and polytype SiC regions with interfaces formed by 30° and 90° extrinsic partial dislocations, as well as zero-Burgers-vector triple dislocation complexes, are expected in the 3C-SiC films. At this, the prevalence of the zero-Burgers-vector complexes is foreseen in view of the smallest distortion introduced into the 3C-SiC lattice.

Results of the MD simulations described here have good agreement with the results of experimental studies [13,14,15,16]. Triple dislocation complexes with the structures HRTEM viewed along the [110] direction, equivalent to those of the zero-Burgers-vector and screw-like complexes shown in the top part of Figure 5, have been demonstrated to form at the interfaces of the 6H-SiC inclusions in cubic silicon carbide [13]. Although the Burgers vectors of these complexes cannot be determined from experimental images without larger-scale $\vec{g}\cdot \vec{b}$ analysis, it is reasonable to attribute these complexes to the stable zero-Burgers-vector triple dislocation complexes discussed here. In [14,15], incoherent twin boundaries of different lengths in CVD-grown 3C-SiC films have been shown to consist of structural units, which are composed of five-, seven-, and six-membered rings (Σ = 3 incoherent twin boundary). The structure of these units resolved by HRTEM in the [110] direction again coincides with the structure of the zero-Burgers-vector (or screw-like) dislocation complex presented in Figure 5.

Lancin et al. [16] used HRTEM to study the atomic structures of the dislocation fronts formed during recrystallization of cubic SiC grains. The authors note that almost no single partial dislocations have been detected, but instead two, three, four, five, and six partial dislocations formed small twin boundaries at the defect fronts. This result is consistent with the high mobilities of single partial dislocations as compared to those of dislocation complexes, as well as the stabilities of the latter, as obtained by the MD simulations. The Burgers vectors for the three- and six-member dislocation fronts have been found equal to zero and their structural units consisted of two 30° and one 90° partials, each having configurations shown in the upper part of Figure 5 [16]. The dislocation fronts consisting of two partials had Burgers vectors corresponding to a 30° partial dislocation and contained one 30° and one 90° dislocations thus forming the structure of the 30° extrinsic dislocation shown in the upper part of Figure 4. The same complexes contributed to the structures of four- and five-dislocation fronts. No 90° extrinsic partial dislocations have been reported. Relative abundance of the three- and six-dislocation front units with zero Burgers vectors has been explained by higher formation probability in view of their lower energy as compared to the energy of the complexes with the Burgers vectors corresponding to the 30° partial dislocations.

## 5. Conclusions

In conclusion, using molecular dynamics simulations with analytical bond order and Vashishta potential, we studied the stability and evolution dynamics of the double and triple partial dislocation complexes in 3C-SiC films. The complexes consisting of PDs with equal Burgers vectors are unstable and dissociate into constituent partial dislocations that tend to separate in view of the repulsive interaction between them. On the other hand, the possibility of the formation of stable dislocation complexes consisting of two (30° and 90° extrinsic partial dislocations) as well as three (zero-Burgers-vector dislocation complex) PDs was demonstrated. The atomic configurations of these complexes do not change during simulated annealing at temperatures up to 1800 K and durations of up to 2 ns. The stability of these complexes originates from the decrease of the dislocation strain energy as a result of complex formation. Moreover, no shift of both double and triple stable dislocation complexes was observed for the simulated annealing temperatures and durations mentioned above, demonstrating their mobility at least orders of magnitude lower than those of single partial dislocations. This indicates that the regions formed by stacking faulted planes in 3C-SiC crystals, such as twin regions and polytype inclusions, should contain the stable complexes of partial dislocations at their interfaces. Such a result is supported by HRTEM experimental observations reported in the literature, in which the formation of twin regions bounded by zero-Burgers-vector complexes and 30° extrinsic partial dislocations as well as 6H-SiC inclusions in 3C-SiC bounded by zero-Burgers-vector complexes have been observed. Furthermore, atomic configurations of the stable PD complexes obtained by MD simulations can be further used for the ab initio study of their electric properties and thus their influence on the electric characteristics of 3C-SiC material.
